# Supplementary material for: Nomogram Predicting the Risk of Postoperative Major Wound Complication in Soft Tissue Sarcoma of the Trunk and Extremities after Preoperative Radiotherapy
Source: Cancers (Basel). 2022 Aug 24;14(17):4096. doi: 10.3390/cancers14174096 (PMC9454623; doi:10.3390/cancers14174096)
Supplement: Supplementary file 1 [file cancers-14-04096-s001.zip › cancers-1865847-supplementary.pdf]

Patient Health Questionnaire (PHQ-9)

Name: ____ Date: ________________________

Over the last 2 weeks, how often have you been bothered by any of the following problems?	Not at all	Several days	More than half the days	Nearly every day
1. Little interest or pleasure in doing things	0	1	2	3
2. Feeling down, depressed, or hopeless	0	1	2	3
3. Trouble falling or staying asleep, or sleeping too much	0	1	2	3
4. Feeling tired or having little energy	0	1	2	3
5. Poor appetite or overeating	0	1	2	3
6. Feeling bad about yourself – or that you are a failure or have let yourself or your family down	0	1	2	3
7. Trouble concentrating on things, such as reading the newspaper or watching television	0	1	2	3
8. Moving or speaking so slowly that other people could have noticed? Or the opposite – being so fidgety or restless that you have been moving around a lot more than usual	0	1	2	3
9. Thoughts that you would be better off dead or of hurting yourself in some way	0	1	2	3

For office coding: Total Score ______ = ______ + ______ + ______

Total Score ______

If you checked off any problems, how difficult have these problems made it for you to do your work, take care of things at home, or get along with other people?

☐ Not difficult at all ☐ Somewhat difficult ☐ Very difficult ☐ Extremely difficult

How to Score the PHQ-9

Major depressive disorder (MDD) is suggested if:

- Of the 9 items, 5 or more are checked as at least 'more than half the days'
- Either item 1 or 2 is checked as at least 'more than half the days'

Other depressive syndrome is suggested if:

- Of the 9 items, between 2 to 4 are checked as at least 'more than half the days'
- Either item 1 or 2 is checked as at least 'more than half the days'

PHQ-9 scores can be used to plan and monitor treatment. To score the instrument, tally the numbers of all the checked responses under each heading (not at all=0, several days=1, more than half the days=2, and nearly every day=3). Add the numbers together to total the score on the bottom of the questionnaire. Interpret the score by using the guide listed below.

**Guide for Interpreting PHQ-9 Scores**		
Score	Depression Severity	Action
0 - 4	None-minimal	Patient may not need depression treatment.
5 - 9	Mild	Use clinical judgment about treatment, based on patient's duration of symptoms and functional impairment.
10 - 14	Moderate	Use clinical judgment about treatment, based on patient's duration of symptoms and functional impairment.
15 - 19	Moderately severe	Treat using antidepressants, psychotherapy or a combination of treatment.
20 - 27	Severe	Treat using antidepressants with or without psychotherapy.

Functional Health Assessment

The instrument also includes a functional health assessment. This asks the patient how emotional difficulties or problems impact work, life at home, or relationships with other people. Patient response of 'very difficult' or 'extremely difficult' suggest that the patient's functionality is impaired. After treatment begins, functional status and number score can be measured to assess patient improvement.

Note: Depression should not be diagnosed or excluded solely on the basis of a PHQ-9 score. A PHQ-9 score $\geq$ 10 has a sensitivity of 88% and a specificity of 88% for major depression.[1] Since the questionnaire relies on patient self-report, the practitioner should verify all responses. A definitive diagnosis is made taking into account how well the patient understood the questionnaire, as well as other relevant information from the patient.

PHQ-9 is adapted from PRIME MD TODAY, developed by Drs Spitzer, Williams, Kroenke and colleagues, with an educational grant from Pfizer Inc.
Use of the PHQ-9 may only be made in accordance with the Terms of Use available at www.pfizer.com. Copyright © 1999 Pfizer Inc. All rights reserved.
PRIME MD TODAY is a trademark of Pfizer Inc.

Reference: Kroenke K, Spitzer RL, Williams JB. The PHQ-9: Validity of a brief depression severity measure. J Gen Intern Med. 2001;16(9):606-613.

GAD-7 Anxiety

Over the <u>last two weeks</u>, how often have you been bothered by the following problems?	Not at all	Several days	More than half the days	Nearly every day
1. Feeling nervous, anxious, or on edge	0	1	2	3
2. Not being able to stop or control worrying	0	1	2	3
3. Worrying too much about different things	0	1	2	3
4. Trouble relaxing	0	1	2	3
5. Being so restless that it is hard to sit still	0	1	2	3
6. Becoming easily annoyed or irritable	0	1	2	3
7. Feeling afraid, as if something awful might happen	0	1	2	3

Column totals _____ + _____ + _____ + _____ =

Total score _______

If you checked any problems, how difficult have they made it for you to do your work, take care of things at home, or get along with other people?

Not difficult at all	Somewhat difficult	Very difficult	Extremely difficult
☐	☐	☐	☐

Source: Primary Care Evaluation of Mental Disorders Patient Health Questionnaire (PRIME-MD-PHQ). The PHQ was developed by Drs. Robert L. Spitzer, Janet B.W. Williams, Kurt Kroenke, and colleagues. For research information, contact Dr. Spitzer at ris8@columbia.edu. PRIME-MD® is a trademark of Pfizer Inc. Copyright© 1999 Pfizer Inc. All rights reserved. Reproduced with permission

Scoring GAD-7 Anxiety Severity

This is calculated by assigning scores of 0, 1, 2, and 3 to the response categories, respectively, of “not at all,” “several days,” “more than half the days,” and “nearly every day.”
GAD-7 total score for the seven items ranges from 0 to 21.

0–4: minimal anxiety

5–9: mild anxiety

10–14: moderate anxiety

15–21: severe anxiety
